# Supplementary material for: The interactome of intact mitochondria by cross-linking mass spectrometry provides evidence for coexisting respiratory supercomplexes
Source: Mol Cell Proteomics. 2017 Dec 8;17(2):216–32. doi: 10.1074/mcp.RA117.000470 (PMC5795388; doi:10.1074/mcp.RA117.000470)
Supplement: Supplemental Data [file supp_RA117.000470_133922_0_supp_23978_fzffwf.zip › spectra_annotation/mito_DR_spectra_annotation/414-1-5-1-4-1.pdf]

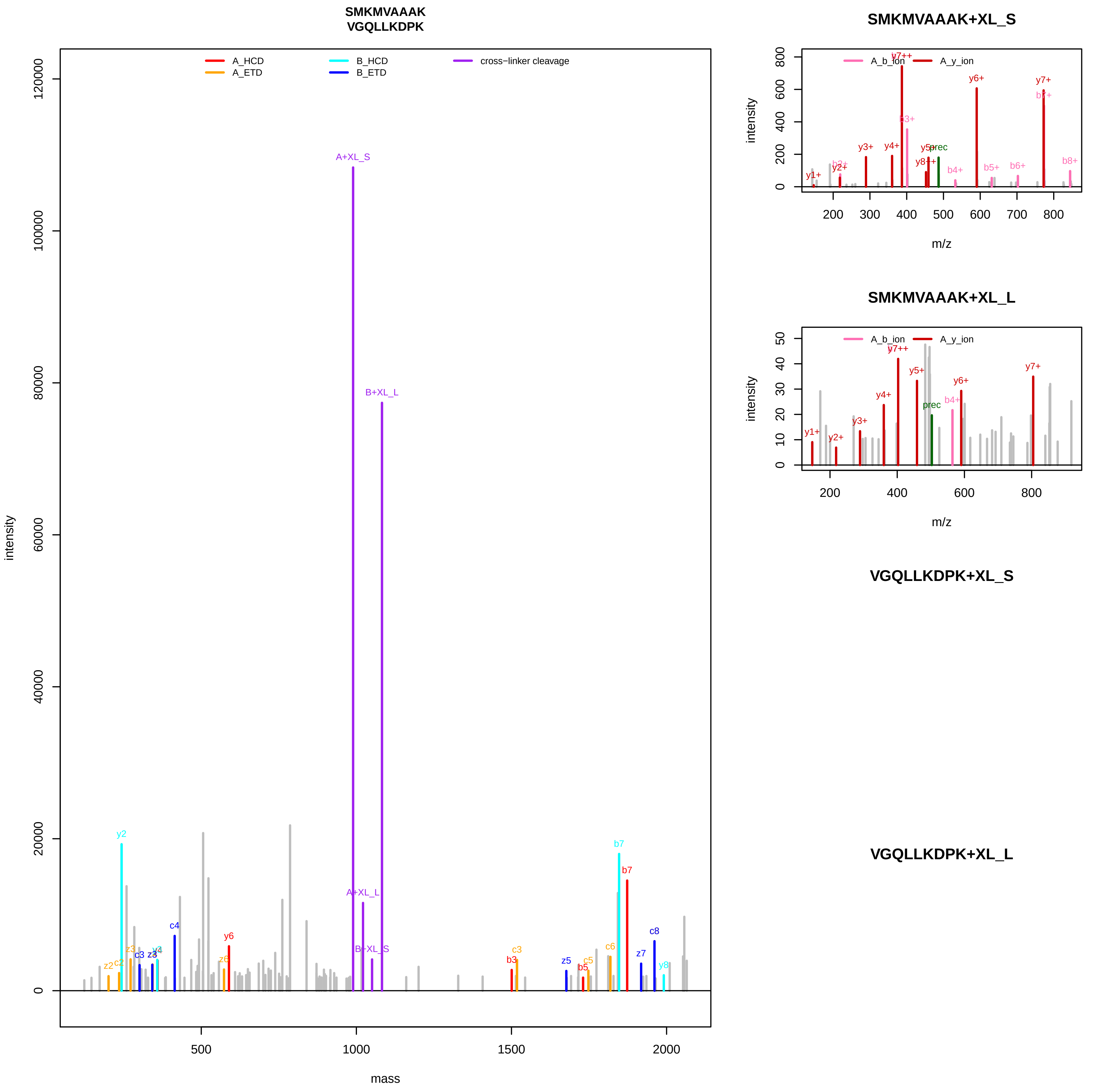

SMKMVAAAK
VGQLLKDPK
A_HCD
A_ETD
B_HCD
B_ETD
cross−linker cleavage
A+XL_S
B+XL_L
A+XL_L
B+XL_S
intensity
mass
y2
b7
b7
c8
y6
c4
z7
y8
z5
b5
c5
c6
b3
c3
z2
c2
z3
c3
z3
y4
z6
0
20000
40000
60000
80000
100000
120000
500
1000
1500
2000
SMKMVAAAK+XL_S
A_b_ion
A_y_ion
y7++
y6+
y7+
b7+
b3+
y3+
y4+
y5+
prec
y8++
y2+
y1+
b4+
b5+
b6+
b8+
intensity
m/z
0
200
400
600
800
200
300
400
500
600
700
800
SMKMVAAAK+XL_L
A_b_ion
A_y_ion
y7++
y5+
y7+
y4+
y6+
prec
b4+
y3+
y1+
y2+
intensity
m/z
0
10
20
30
40
50
200
400
600
800
VGQLLKDPK+XL_S
VGQLLKDPK+XL_L
